# Supplementary material for: COVID-19 vaccine access and attitudes among people experiencing homelessness from pilot mobile phone survey in Los Angeles, CA
Source: PLoS One. 2021 Jul 30;16(7):e0255246. doi: 10.1371/journal.pone.0255246 (PMC8323924; doi:10.1371/journal.pone.0255246)
Supplement: Attachment — Submitted filename: Response to Reviewers 20210514.docx [file pone.0255246.s002.docx]

|  |  |  |
| --- | --- | --- |
|  |  | 650 Charles E. Young Dr. South  36-071 CHS, Box 951772  Los Angeles, CA 90095-1772 |
|  | | |

May 19th, 2021

PLOS ONE

To the Editor and Reviewers:

Thank you for your response and feedback to our submission “COVID-19 vaccine access and attitudes among people experiencing homelessness from pilot mobile phone survey in Los Angeles, CA.”

We have expanded the discussion to address the points raised by Reviewer #1, namely: we expanded upon the clinical implications of the study, specifically noting the historic impact of communicable diseases within homeless encampments and shelters, and the importance of vaccine acceptance beyond COVID-19 (see lines 247-255). We also addressed the global implications of our findings (see lines 245-247), in drawing parallels to people experiencing homeless with other vulnerable populations around the world.

We also addressed the following points raised by the academic editor:

- Ensured our manuscript meets PLOS ONE’s style requirements including those for file naming
- Published our minimal data set underlying the results
- Revised our funding statement to address our affiliation with Akido Labs: *“Funding for this study was provided by University of California Office of the President Emergency COVID-19 Research Seed Funding Grant #R00RG2888 “A data-driven response to coronavirus among homeless clients in LA County” and the USC Homeless Policy Research Institute. These funders provided support in the form of salaries for authors AL, MK and KM but did not have any additional role in the study design, data collection and analysis, decision to publish, or preparation of the manuscript. The specific roles of these authors are articulated in the ‘author contributions’ section.”*
- Revised our competing interests statement to address our affiliation with Akido Labs: *“The authors have declared that no competing interests exist. Akido Labs is a commercial technology and data management company. The research team for this project contracted with Akido as a technology partner to support survey design and distribution, data acquisition, critical revision of the manuscript, and administrative and technical support. This does not alter our adherence to PLOS ONE policies on sharing data and materials”*
- Expanded our reference list (added #18-21) to cite the points that were made in response to Reviewer #1’s requested changes

Sincerely,

Randall Kuhn

Associate Professor, Department of Community Health Sciences

UCLA
